# Clinical Features and Correlates of Poor Nighttime Sleepiness in Patients with Parkinson's Disease

**DOI:** 10.1155/2020/6378673

**Published:** 2020-09-14

**Authors:** Xiaoling Qin, Xue Li, Gang Chen, Xu Chen, Mingyu Shi, Xue-kui Liu, Zai-li Li, Zai-e Xin, Dianshuai Gao

**Affiliations:** ^1^Department of Neurology, Shanghai 8^th^ People's Hospital, Affiliated to Tongji University, No. 8 Caobao Road, Shanghai 200235, China; ^2^Xuzhou Key Laboratory of Neurobiology, Department of Neurobiology and Anatomy, Xuzhou Medical University, Xuzhou 221004, Jiangsu, China; ^3^Xuzhou Clinical School of Xuzhou Medical University, No. 199 Jiefang Road, Xuzhou 221009, Jiangsu, China

## Abstract

**Objective:**

The present study investigated the clinical features and correlates of poor nighttime sleepiness (PNS) in patients with Parkinson's disease (PD).

**Methods:**

One hundred ten patients with PD (divided into PD-PNS group and PD-nPNS group) and forty-seven controls (nPD-PNS group) were enrolled in this study. Demographic information was collected. Patients were assessed according to the unified Parkinson's disease rating scale (UPDRS) and Hoehn–Yahr (H&Y) stage scale. Patients were also evaluated according to the Pittsburgh sleep quality index (PSQI), Epworth sleepiness scale (ESS), rapid eye movement sleep behavior disorder screening questionnaire (RBD-SQ), restless leg syndrome (RLS) diagnosis, Hamilton's depression scale (HAMD), and Hamilton's anxiety scale (HAMA).

**Results:**

The prevalence of PNS was 55.45% (61/110) in patients with PD. The PD-PNS group tended to have a longer duration of disease, higher UPDRS-I and UPDRS-III scores, a higher percentage of RLS patients, and higher HAMA and HAMD scores than those of the PD-nPNS group. The PD-PNS group tended to have a higher percentage of RBD and RLS patients and higher HAMA and HAMD scores than those of the nPD-PNS group. Analysis of the PSQI components and PSQI impact factors showed that the PD-PNS group had worse subjective sleep quality (*χ*^2^ = −2.267, *P* = 0.023), shorter sleep latency (*χ*^2^ = −2.262, *P* = 0.024), fewer sleep medications (*χ*^2^ = −4.170, *P* ≤ 0.001), worse daytime functioning (*χ*^2^ = −2.347, *P* = 0.019), and an even higher prevalence of increased nocturia (*χ*^2^ = 4.447, *P* = 0.035), nightmares (*χ*^2^ = 7.887, *P* = 0.005), and pain (*χ*^2^ = 9.604, *P* = 0.002) than those of the nPD-PNS group. Analysis also indicated that the PSQI global score positively correlated with BMI (*r* = 0.216, *P* < 0.05), H&Y stage (*r* = 0.223, *P* < 0.05), UPDRS-I (*r* = 0.501, *P* < 0.01), UPDRS-III (*r* = 0.425, *P* < 0.01), ESS (*r* = −0.296, *P* < 0.01), RBD (*r* = 0.227, *P* < 0.05), RLS (*r* = 0.254, *P* < 0.01), HAMA (*r* = 0.329, *P* < 0.01), and HAMD (*r* = 0.466, *P* < 0.01). In the final model, H&Y stage, RLS, UPDRS-III, and HAMD remained associated with the PQSI score (*P* ≤ 0.001, *P* ≤ 0.001, *P* = 0.049, *P* ≤ 0.001, respectively).

**Conclusions:**

Our data showed that PNS was common in patients with PD. H&Y stage, UPDRS-III, HAMD, and RLS were positively associated with PNS. Attention to the management of motor symptoms, RLS, and depression may be beneficial to nighttime sleep quality in patients with PD.

## 1. Introduction

Parkinson's disease (PD) is the second most common neurodegenerative disease and is characterized by motor and nonmotor dysfunctions [1]. Disordered sleep is one of the most frequent nonmotor symptoms in PD patients and has a significant negative impact on quality of life [2]. Disordered sleep affects 40–98% of PD patients worldwide and 47.66–89.10% of PD patients in China [3, 4]. The possible pathogenesis of PD with sleep disturbance includes thalamocortical pathway degeneration and changes in neurotransmitter systems [5]. The etiology of sleep disturbance is multifactorial and involves the degeneration of areas regulating sleep, sleep structure affected by drugs, drug-induced sleep disturbance, and sleep fragmentation due to multiple factors [6]. Although many studies performed over the past decade have investigated the clinical characteristics of sleep disturbance among patients with PD, some issues remain unclear and warrant further delineation.

Sleep disorders in PD patients include insomnia, vivid dreams, restless legs syndrome (RLS), rapid eye movement sleep behavior disorder (RBD), periodic limb movements (PLM), circadian rhythm disruption, and excessive daytime sleepiness (EDS), which lead to nighttime and daytime sleep problems [7]. Nighttime and daytime sleep problems significantly impair quality of life, especially nighttime sleep problems, which increase the risk of cardiovascular and cerebrovascular events and lead to an economic burden for patients and their caregivers [8]. Thus, it is important to identify those patients with nighttime sleep problems early and provide potential treatment options as soon as possible. Identifying patients with unique clinical features can facilitate analyses of subtype specific biomarkers and epidemiological and clinical treatments. So far, few studies have focused on overall nighttime sleep quality in Chinese patients with PD, and clinical features and correlates of poor nighttime sleepiness in PD patients have not been investigated.

Currently, objective assessment of sleep physiology relies primarily on polysomnography (PSG), which records the electroencephalographic (EEG) activity, extraocular eye movements, mentalis muscle tone, air flow, respiratory effort, and cardiac rhythm to determine sleep staging and aids in diagnosing different types of sleep disorders. However, the assessment of sleep quality is usually based on patient self-reporting, interviews, and psychological variables. The latter can better reflect the subjective feelings of patients and directly represent clinical symptoms, which help researchers to easily obtain clinical characteristics and quickly screen target patients. The Pittsburgh sleep quality index (PSQI) is generally used to evaluate overall nighttime sleep quality and widely applied in clinical work and clinical research. Patients with PSQI scores higher than 7 are generally considered poor nighttime sleep (PNS) patients [9].

In general population, self-reported sleep disturbances are increasingly common with advancing age. In Outcomes of Sleep Disorders in Older Men (MrOS Sleep) study, up to 40.7% of older community-dwelling men were reported to have poor nighttime sleep quality (PSQI > 5) [10]. Additionally, age-related changes in sleep/wake patterns including lower sleep efficiency, longer sleep latency, greater nighttime wakefulness, and higher number of long wake episodes have been reported in population-based cohorts of older men and women [11]. Prior population-based studies in older people have also reported a high prevalence of sleep-disordered breathing and periodic leg movements in sleep [12]. To our knowledge, there are few studies investigating the nighttime sleep patterns of PD patients or discussing the differences of clinical features in sleep disorders that may exist between PD patients and general populations. The present study therefore aimed to characterize the clinical features of PNS in patients with PD and control subjects, to investigate the correlates of PNS among PD patients.

## 2. Methods

### 2.1. Patients

A total of 110 PD patients were recruited from the clinic or impatient Department of Neurology, Xuzhou Central Hospital/Clinical Hospital of Xuzhou Medical University, from March 2017 to July 2018. The clinical diagnosis of idiopathic PD was determined based on the MDS clinical diagnostic criteria for Parkinson's disease [13]. Forty-nine simple poor nighttime sleepers without PD were recruited from the clinic and selected as controls. People with other diseases, such as respiratory diseases, urinary system diseases, cardiovascular and cerebrovascular diseases, and primary mental disorders, were excluded. People who were unable to finish the questionnaire were also excluded.

The Ethics Committee of Xuzhou Clinical Hospital of Xuzhou Medical University approved this study.

### 2.2. Assessments

Each participant completed the Pittsburgh sleep quality index (PSQI), Epworth sleepiness scale (ESS), rapid eye movement sleep behavior disorder screening questionnaire (RBD-SQ), restless leg syndrome (RLS) diagnosis, Hamilton's depression scale (HAMD), and Hamilton's anxiety scale (HAMA). The PSQI is a self-report questionnaire that assesses nighttime sleep over a 1-month time period [9]. Nineteen individual items generate seven “component scores”: subjective sleep quality, sleep latency, sleep duration, habitual sleep efficiency, sleep disturbances, sleep medication, and daytime dysfunction. The scores for these components range from 0 (no difficulty) to 3 (severe difficulty) and are summed to produce a global measure of sleep disturbance, with a global score ranging from 0 to 21. Higher scores represent poorer subjective sleep quality. Notably, the fifth item is composed of 9 nighttime symptoms that may affect sleep quality, including difficulty falling asleep, fragmented sleep or awakening earlier, increased nocturia, disturbance in respiration, cough or snoring, feeling cool, feeling hot, nightmares, and pain. Subjects in the present study with PSQI > 7 were considered poor nighttime sleepers [14]. According to their PSQI scores, PD subjects were divided into PD-PNS group (PSQI > 7) and PD-nPNS group (PSQI < 7). Subjects with PSQI > 7 but without Parkinson's disease were selected for the nPD-PNS group.

A movement disorder specialist clinically evaluated PD subjects in an “on” state. Clinical data were collected, including demographic information (age, sex, education), age of movement symptom onset, disease duration, concurrent diseases, medication information for PD (the levodopa-equivalent dose (LED) was calculated based on previously reported conversion factors) [15], and detailed medical history. The unified Parkinson's disease rating scale (UPDRS) and Hoehn–Yahr stage (H&Y stage) scale were applied to all PD subjects in an “on” state [16]. Motor phenotypes were identified based on the ratio of the mean tremor score (sum of items 20 and 21 in the UPDRS-III divided by four) to the mean bradykinesia/rigid score (sum of items 22–27 and 31 in the UPDRS-III divided by 15). Patients with a ratio greater than 1.0, less than 0.80, and between 0.80 and 1.0 were classified into the tremor-dominant (TD) subtype, akinetic-rigid (AR) subtype, and mixed subtype, respectively [17].

All subjects underwent evaluations using the ESS, rapid eye movement (REM) RBD-SQ, RLS diagnosis, HAMD, and HAMA. The ESS is a widely used questionnaire for assessing the general level of daytime sleepiness. The ESS is composed of eight items that address typical day-to-day situations. Each item ranges from 0 to 3 points (0 = would never doze, 3 = high chance of dozing) to yield a total ESS score of 0–24 (lowest to highest sleep propensity). Subjects with ESS > 10 were considered excessive daytime sleepers (EDS), and normal sleep propensity was 0–10 [18]. The RBD-SQ is a valuable tool for screening rapid eye movement (REM) sleep behavior disorder (RBD) [19]. An RBD score of 5 or greater was defined as probable RBD. A diagnosis of RLS was made according to the RLS diagnostic criteria proposed by the International Restless Legs Syndrome Study Group (IRLSSG) in 2014, which is based on four essential features of the questionnaire after the exclusion of RLS mimics, such as positional discomfort, muscle cramp, venous stasis, vascular claudication, and peripheral neuropathy [20]. HAMD and HAMA were used to assess depression and anxiety, respectively [21]. Neurologists confirmed the final results.

### 2.3. Statistical Analysis

The measurement data are expressed as means ± SD (standard deviation), and the enumeration data are shown as numbers (rate). Two independent sample *t*-tests were used to analyze the measurement data of two groups with a normal distribution. Non-normal distribution data were analyzed using nonparametric tests (Mann–Whitney test). The enumeration data were analyzed using the *χ*^2^ test. We used Spearman's correlation to assess the correlations between the different factors and PSQI global score and PSQI components. The PSQI global score was the dependent variable, and clinical factors were independent variables. Line regression analysis was used to show the relationship of the parameters. *P* < 0.05 was considered statistically significant.

## 3. Results

### 3.1. Demographic and Medication Data of PD Patients with and without PNS

A total of 110 PD patients were included in this study. The demographic data and medication data of PD patients with PNS (PD-PNS) and without PNS (PD-nPNS) are shown in Table 1.

Sixty-one of the 110 PD patients were included in PD-PNS group, and the prevalence of PNS was 55.45% (61/110). For all the PD-PNS subjects, the percentages of male and female patients were 47.54% (29/61) and 44.9% (23/49), respectively. The mean age of PD-PNS patients was 66.57 ± 8.73 years. The average age of PD onset and disease duration was 60.76 ± 10.18 years and 48 (17–96) months, respectively. The mean years of education were 8.34 ± 1.16 years. The average BMI was 23.00 ± 3.42 kg/m^2^. The mean daily LED of the patients in our study was 431.93 ± 219.76 mg/day. The average H&Y stage was 2 (1.5–3.0). The average UPDRS-I and UPDRS-III scores were 10 (4–16) and 23 (14–41), respectively. The mean ESS score was 5 (1–11.75). The percentages of RBD- and RLS-positive patients were 36.07% (22/61) and 33.79% (20/61), respectively. The average HAMA scores and HAMD scores were 3.5 (1–8) and 4.5 (2.75–13), respectively.

The other 49 cases were included in the PD-nPNS group. The percentages of male and female patients were 55.1% (27/49) and 52.46% (32/61), respectively. The mean age of PD-nPNS patients was (67.12 ± 7.63) years. The average age of PD onset and disease duration were 61.85 ± 10.70 years and 36 (12–51) months, respectively. The mean years of education were 9.11 ± 0.80 years. The average BMI was 23.40 ± 3.34 kg/m2. The mean daily LED of the patients in our study was 466.48 ± 243.82 mg/day. The average H&Y stage was 2 (1.5–2.0). The mean ESS score was 4.0 (1.0–8.0). The percentages of RBD- and RLS-positive patients were 20.43% (10/49) and 12.24% (6/49), respectively. The average HAMA and HAMD scores were 1 (0–5.25) and 1.5 (0.75–6.25), respectively.

The PD-PNS group tended to have a longer duration of disease, higher UPDRS-I and UPDRS-III scores, a higher percentage of RLS patients, and higher HAMA and HAMD scores than those the PD-nPNS group. However, there were no significant differences in age, sex, educational year, BMI, age of onset, daily LED, H&Y stage, ESS scores, or percentage of RBD patients between the two groups.

### 3.2. Demographic and Medication Data of PNS with or without PD

The demographic and medication data of PNS patients with PD (PD-PNS) and without PD (nPD-PNS) are shown in Table 2.

The data from the PD-PNS group are described above. Forty-seven cases were included in the nPD-PNS group. The percentages of male and female patients were 53.19% (25/47) and 46.81% (22/47), respectively. The mean age of nPD-PNS patients was 63.96 ± 10.51 years. The mean years of education were 9.46 ± 1.15 years. The average BMI was 23.52 ± 2.98 kg/m2. The mean ESS score was 3 (1–9). The percentages of RBD- and RLS-positive patients were 4.30% (2/47) and 6.4% (3/47), respectively. The average HAMA and HAMD scores were 4 (2–8) and 3 (2–6.25), respectively.

There were no significant differences in the PSQI global scores, age, sex, educational year, or BMI between PD-PNS group and nPD-PNS group. Therefore, the ESS, HAMA, and HAMD scores and the percentages of RBD and RLS patients were statically compared between the two groups. The PD-PNS group tended to have higher percentages of RBD and RLS patients and higher HAMA and HAMD scores than those of the nPD-PNS group. However, there was no significant difference in ESS scores between the two groups.

The seven items of the PSQI components were statistically compared. According to the K–S test, the score of each of the 7 factors in the PSQI was skewed, although the global score of the PSQI was normally distributed. Therefore, *t*-tests and nonparametric tests were performed. The final results indicated that subjective sleep quality (*χ*^2^ = −2.267, *P* = 0.023), sleep latency (*χ*^2^ = −2.262, *P* = 0.024), use of sleeping medication (*χ*^2^ = *P*4.170, *P* ≤ 0.001), and daytime dysfunction (*χ*^2^ = *P*2.347, *P* = 0.019) were significantly different between the PD-PNS group and nPD-PNS group. The PD-PNS group had worse subject sleep quality (*χ*^2^ = −2.267, *P* = 0.023), shorter sleep latency (*χ*^2^ = −2.262, *P* = 0.024), fewer sleep medications (*χ*^2^ = −4.170, *P* ≤ 0.001), and worse daytime function (*χ*^2^ = −2.347, *P* = 0.019), and the nPD-PNS group exhibited the opposite effects. There was no significant difference in sleep duration (*χ*^2^ = −0.027, *P* = 0.979), sleep efficiency (*χ*^2^ = −0.322, *P* = 0.748), or sleep disturbances (*χ*^2^ = −0.442, *P* = 0.658) between the two groups.

We also compared the differences in the prevalence of the 9 nighttime symptoms between the two groups. The PD-PNS group had higher prevalence of increased nocturia (*χ*^2^ = 4.447, *P* = 0.035), nightmares (*χ*^2^ = 7.887, *P* = 0.005), and pain (*χ*^2^ = 9.604, *P* = 0.002). There were no significant differences in difficulty falling asleep (*χ*^2^ = 1.345, *P* = 0.246), fragmented sleep (*χ*^2^ = 0.011, *P* = 0.918), disturbance sin respiration (*χ*^2^ = 0.095, *P* = 0.758), feeling cool (*χ*^2^ = 0.062, *P* = 0.803), or feeling hot (*χ*^2^ = 1.542, *P* = 0.241) between the two groups.

### 3.3. Correlations of PSQI Global Score or PSQI Components with Other Factors in PD-PNS Patients

Table 3 shows the factors associated with PNS. Pearson's or Spearman's correlation analysis was performed based on the normal or skewed distribution of the data, respectively. The analysis indicated that the PSQI global score positively correlated with BMI (*r* = 0.216, *P* < 0.05), H&Y stage (*r* = 0.223, *P* < 0.05), UPDRS-I (*r* = 0.501, *P* < 0.01), UPDRS-III (*r* = 0.425, *P* < 0.01), ESS (*r* = −0.296, *P* < 0.01), RBD (*r* = 0.227, *P* < 0.05), RLS (*r* = 0.254, *P* < 0.01), HAMA (*r* = 0.329, *P* < 0.01), and HAMD (*r* = 0.466, *P* < 0.01). UPDRS I, ESS, HAMA, and HAMD were the most prominent factors that correlated with all of the 7 PSQI components, except sleep medication (*P* < 0.01).

### 3.4. Regression Analysis of Factors Associated with the PSQI Global Score of PD-PNS Patients

Using the PSQI global score as the dependent variable and sex, age, disease duration, BMI, LED, H&Y stage, UPDRS I, UPDRS III, ESS, RBD, RLS, HAMA, and HAMD as independent variables, we performed stepwise linear regression analysis to identify the risk factors of the PSQI global score. The results showed that the included variables H&Y, UPDRS-III, RLS, and HAMD positively correlated with the PSQI global score (*P* ≤ 0.001, *P* ≤ 0.001, *P* = 0.049, and *P* ≤ 0.001, respectively), indicating that they are positive risk factors for the dependent variable. The *R*^2^ was 0.577, and the adjusted *R*^2^ was 0.541 in the model of regression (Table 4).

## 4. Discussion

The present cross-sectional study investigated the prevalence, clinical characteristics, and associated factors of PNS in PD patients and control subjects in a Chinese population. A wide range of evaluation methods were used to assess various factors that potentially influence PNS. Our findings did not show significant differences in demographic factors but showed significant differences in clinical symptoms between PD patients with and without PNS, indicating that PNS was associated with many variables. We also confirmed significantly higher prevalence of RBD, RLS, and depression in PD-PNS subjects than in age-matched and sex-matched nPD-PNS subjects. The findings also showed significant differences in clinical symptoms between PNS patients with and without PD.

The prevalence of PNS was 55.45% in patients with PD in the present study, which is higher than that reported in other studies [22]. This difference may be attributed to differences in the methodology and questionnaire interpretation. PD-PNS subjects showed specific clinical parameters. PD-PNS subjects tended to have higher UPDRS-I, UPDRS-III, HAMA, and HAMD scores compared with those of PD-nPNS subjects. UPDRS-I and UPDRS-III represent disease-related psychological conditions and the disability of PD subjects, respectively. These findings suggest that mental and motor impairment, together with anxiety and depression, are associated with PD-PNS.

RLS is a common disorder characterized by a convincing urge to move the lower limbs accompanied by unpleasant sensations and symptoms that are aggravated during rest and alleviated by activity [23]. The association between RLS and Parkinson's disease (PD) is not clear, but dopaminergic hypofunction in the central nervous system is present in both diseases [24, 25]. The prevalence of RLS in PD patients ranges from 8.41% to 34.85% in China and is about 15% in the world. The present study found that 23.64% of total PD patients had RLS, which is consistent with a previous study [23, 26]. The PD-PNS group included a significantly higher percentage of RLS patients (33.79%) than PD-nPNS group (12.24%), which indicates that RLS affects the quality of nighttime sleep in PD patients.

The prevalence of RLS in the general adult population in Asia is reported to be 1–4% [27, 28], compared to approximately 7–10% in Europe and the United States [17]. Although RLS is a possible preclinical marker of PD or an early clinical feature of PD, a recent study has shown that the prevalence of RLS in PD patients is not significantly different from the general population, which contradicts the epidemiological association [25]. However, in our study, the proportion of RLS patients in PD-PNS group (33.79%) was significantly higher than nPD-PNS group (6.4%), indicating that these two PNS groupsmay not share the same mechanism, supporting the difference in RLS between PD patients and general populations.

RBD is characterized by the loss of normal muscle atonia during REM sleep. Patients often experience violent dream-enacting behaviors, which leads to disturbed sleep and potential injuries to themselves and their bed partners. Polysomnography (PSG) remains the diagnostic gold standard, but the diagnosis of probable RBD may be made based on clinical judgment or validated questionnaires [7]. RBD is an early sign of neurodegenerative disease, with a conversion rate from RBD to PD of 18.6–65.0% [29]. The association between PD and RBD can be explained as neurodegeneration in certain brainstem structures at Braak stage 1-2 [23]. It was reported that the prevalence of RBD in PD patients ranges from 22.2% to 60.0% in China [24]. The prevalence of RBD in the entire sample of PD patients in the present study was 27.27%, as judged by the RBD-SQ. There was no significant difference in the prevalence of RBD between PD-PNS and PD-nPNS groups, but significant differences between the PD-PNS and nPD-PNS groups were observed. RBD correlated with the PSQI global score and sleep disturbance, but it was not mainly associated with the PSQI global score in the final model. Therefore, RBD is more likely to occur in PD patients and affect those patients' nighttime sleep.

Excessive daytime sleepiness (EDS) was defined as an inability to maintain wakefulness and alertness during the major waking episodes of the day that resulted in periods of an irrepressible need for sleep or unintended lapses into drowsiness or sleep [7]. The present study used ESS to evaluate and calculate the prevalence of EDS. There were no significant differences between PD-PNS and PD-nPNS groups or PD-PNS and nPD-PNS groups. Other studies reported that the prevalence of EDS was higher in PD patients than in the general population [30, 31]. The present study finally indicated that ESS was associated with PQSI scores and some of PQSI components, including subjective sleep, sleep duration, sleep efficiency, and sleep disturbance, but was not the main factor associated with PNS in PD patients.

Multiple regression analysis showed that H&Y stage, UPDRS-III, RLS, and HAMD were the main factors associated with PNS in PD patients. These findings suggested that the disease severity, motor impairments, RLS, and depression were the main factors affecting nighttime sleep quality of patients with PD. PD-PNS group had significantly higher UPDRS-Iand UPDRS-III scores, especially UPDRS-III, which represents the severity of motor symptoms. UPDRS-III was one of the main factors associated with PQSI scores in the final analysis, suggesting that motor symptoms have a significant positive effect on PNS; the worse the motor symptoms, the worse the nighttime sleep quality. Motor symptoms have been considered to be related to sleep disorders and cause nocturnal problems in other studies [32]. In a study by Suzuki et al., patients with PD-related sleep disorders had higher MDS-UPDRS part II, III, and IV scores. Notably, they found a significant link between the clinical motor subtypes (tremor dominant or postural instability and gait difficulty) and sleep-related symptoms [32].

RLS, correlating with the PSQI global score and daytime dysfunction in the present study, was also one of the main factors associated with the PSQI global score. PD patients with RLS exhibited higher PSQI score with poorer daytime dysfunction than PD patients without RLS and tended to manifest PNS. However, we did not find impacts of RBD and EDS on PNS in this study or find the difference in prevalence of RBD and EDS between the PD-PNS and PD-nPNS group. This result is consistent with other studies [33, 34]. Some studies pointed out that treatment for RLS could improve sleep quality and quality of life [35]. However, in other studies, the results have been contradictory or complementary [36]. In another study, researchers believed that RLS had less impact on sleep problems than EDS and RBD, and they explained that the results were influenced by the range of severity of RLS [32].

Among the psychiatric symptoms of PD, depression is the most common, followed by anxiety, or both coexisting. HAMD and HAMA were applied to assess depression and anxiety, respectively, in this study. We found that patients in PD-PNS group had significantly higher HAMD and HAMA scores than PD-nPNS group. In PD-PNS group, only the HAMD scores were significantly higher than those of nPD-PNS group. Both HAMD and HAMA were correlated with PSQI global score and subjective sleep, sleep latency, sleep latency, sleep efficiency, sleep disturbance, and daytime dysfunction. In the present study, final analysis revealed that depression, rather than anxiety, was one of the three main factors significantly associated with PNS. Previous studies have also found that depression or/and anxiety are important risk factors for poor sleep quality in patients with PD [37, 38]. Contrary to our results, however, Menza and Rosen reported that depression did not significantly affect any of the sleep quality variables [39].

In addition, we found that PNS subjects had lower subjective sleep quality, shorter sleep latency, poorer daytime function, and fewer sleep medications. Night sleep quality in patients with PD may be affected by three main symptoms, namely, increased nocturia, nightmares, and pain. These symptoms are also common nonmotor symptoms in patients with PD [40], suggesting that some nonmotor symptoms may affect the quality of nighttime sleep. Similar views have been noted in other studies that have shown significant correlations between nonmotor symptoms and PD-related sleep problems in PD patients but have not emphasized nighttime sleep problems [41].

In summary, PNS is common in patients with PD, with three main risk factors of severe motor symptoms, RLS, and depression. The differences in sleep pattern between PD-PNS and nPD-PNS patients spotlighted the fact that PD-PNS subjects had higher prevalence of RBD and RLS, higher scores of HAMD, lower subjective sleep quality, shorter sleep latency, poorer daytime function, and fewer sleeping medication. To our knowledge, the present study is a rare attempt to characterize PNS in patients with PD and to differentiate PNS between PD patients and the general population.

Considering the impact of PNS on patients' quality of life, physicians caring for PD patients should pay attention to nighttime sleep quality and actively prescribe appropriate medications for treatment, especially in patients with RLS, severe motor symptoms, and higher HAMD scores, who are at high risk for PNS.

### 4.1. Limitation

Our study was designed as a questionnaire-based interview investigation. Although it had its own advantages as we mentioned in the manuscript, it is to some degree lacking in strong objective data such as polysomnography or mean sleep latency tests, which limited the objectivity and accuracy of the findings. In addition, patients needed enough mental and physical condition to complete the questionnaires.

## Figures and Tables

**Table 1:** Demographic and medication data of PD patients with or without PNS.

	PD (*n* = 110)		*t*/*Z*/*χ*^2^	*P*
	PD-PNS (*n* = 61)	PD-nPNS (*n* = 49)		
Age (*y*)	66.57 ± 8.73	67.12 ± 7.63	1.458	0.622
Sex, female (%)	29 (47.54)	27 (55.1)	0.838	0.360
Education (*y*)	8.34 ± 1.16	9.11 ± 0.80	3.451	0.178
BMI	23.00 ± 3.42	23.40 ± 3.34	0.471	0.275
Disease duration (*m*)	48 (17–96)	36 (12–51)	−1.698	0.116
Age of onset	60.76 ± 10.18	61.85 ± 10.70	−0.867	0.388
Family history				
PD family history	0 (0)	0 (0)	—	—
ET family history	1 (1.45%)	0 (0)	—	—
Phenotype				
TD	16 (26.23%)	14 (28.57%)	0.835	0.659
AR	25 (40.98%)	16 (32.65%)		
Mixed	20 (32.79%)	19 (38.78%)		
H&Y (onstage)	2 (1.5–3.0)	2 (1.5–2.0)	−0.989	0.323
UPDRS-I	10 (4–16)	6 (3–7)	−2.742	0.006^*∗∗*^
UPDRS-III	23 (14–41)	12 (9–27)	−2.709	0.007^*∗∗*^
LED (mg)	431.93 ± 219.76	466.48 ± 243.82	0.611	0.533^*∗∗*^
ESS (scores)	5 (1–11.75)	4.0 (1.0–8.0)	−1.458	0.145
RBD, case (%)	22 (36.07)	10 (20.43)	2.948	0.086
RLS, case (%)	20 (33.79)	6 (12.24)	6.420	0.011^*∗*^
HAMA (scores)	3.5 (1–8)	1 (0–5.25)	−2.452	0.014^*∗*^
HAMD (scores)	4.5 (2.75–13)	1.5 (0.75–6.25)	−3.651	≤0.001^*∗∗*^
PSQI global score	13 (10–15)	2 (1–4)	—	—
PSQI components (scores)				
Subjective sleep quality	2 (1–3)	0 (0–0.25)	—	—
Sleep latency	2 (1–3)	0 (0–1)	—	—
Sleep duration	3 (2–3)	0 (0–0.25)	—	—
Sleep efficiency	3 (3–3)	0 (0–0.25)	—	—
Sleep disturbances	1 (1–2)	1 (0–1)	—	—
Sleeping medication	0 (0–0)	2 (0–3)	—	—
Daytime dysfunction	3 (1–3)	1 (0–2)	—	—
PSQI impact factors, case (%)				
Difficulty falling asleep	37 (60.67)	3 (6.12)	—	—
Fragment sleep	39 (63.94)	7 (14.29)	—	—
Increased nocturia	30 (50.82)	12 (24.49)	—	—
Disturbance in respiration	3 (4.92)	0 (0)	—	—
Cough or snoring	13 (19.67)	7 (14.29)	—	—
Feeling cool	2 (3.29)	0 (0)	—	—
Feeling hot	10 (16.39)	1 (2.04)	—	—
Nightmares	12 (19.67)	6 (12.24)	—	—
Pain	11 (18.03)	1 (2.04)	—	—

PD: Parkinson's disease; PNS: poor nighttime sleep; PD-PNS: PD with poor nighttime sleep; PD-nPNS: PD without poor nighttime sleep; BMI: body mass index; ET: essential tremor; LED: daily levodopa-equivalent dose; TD: tremor-dominant subtype; AR: akinetic-rigid subtype; mixed: mixed subtype; H&Y: Hoehn–Yahr stage; UPDRS-I: united Parkinson's disease ranking scale part-I; UPDRS III: united Parkinson's disease ranking scale part- III; PSQI: Pittsburgh sleep quality index; ESS: Epworth sleepiness scale; RBD: rapid eye movement (REM) sleep behavior disorder; RLS: restless legs syndrome; HAMA: Hamilton's anxiety scale; HAMD: Hamilton's depression scale; ^*a*^Mann–Whitney test; ^*b*^*t*-test; ^*∗*^*P* < 0.05; ^*∗∗*^*P* < 0.01.

**Table 2:** Demographic and medication data of PD-PNS and nPD-PNS groups.

	PD-PNS (*n* = 61)	nPD-PNS (*n* = 47)	*Z* value (*χ*^2^)	*P*
Age (*y*)	66.57 ± 8.73	63.96 ± 10.51	2.503	0.209
Sex, female (%)	29 (47.54)	25 (53.19)	0.449	0.503
Education (*y*)	8.34 ± 1.16	9.46 ± 1.15	0.719	0.568
BMI	23.00 ± 3.42	23.52 ± 2.98	2.571	0.173
ESS (scores)	5 (1–11.75)	3 (1–9)	−1.644	0.100
RBD, case (%)	22 (36.07)	2 (4.3)	15.912	≤0.001^*∗∗*^
RLS, case (%)	20 (33.79)	3 (6.4)	11.344	0.001^*∗∗*^
HAMA (scores)	3.5 (1–8)	4 (2–8)	−0.473	0.636
HAMD (scores)	4.5 (2.75–13)	3 (2–6.25)	−2.275	0.023^*∗*^
PSQI global score	13 (10–15)	14 (11–17)	−1.833	0.067
PSQI components (scores)				
Subjective sleep quality	2 (1–3)	3 (1–3)	−2.267	0.023^*∗*^
Sleep latency	2 (1–3)	3 (1–3)	−2.262	0.024
Sleep duration	3 (2-3)	3 (2–3)	−0.027	0.979
Sleep efficiency	3 (3–3)	3 (3–3)	−0.322	0.748
Sleep disturbances	1 (1–2)	1 (1–2)	−0.442	0.658
Sleep medication	0 (0–0)	2 (0–3)	−4.170	≤0.001^*∗∗*^
Daytime dysfunction	3 (1–3)	1 (1-2)	−2.347	0.019^*∗*^
PSQI impact factors, case (%)				
Difficulty falling asleep	37 (60.67)	34 (72.3)	1.345	0.246
Fragment sleep	39 (63.94)	31 (66.0)	0.011	0.918
Nocturia increased,	30 (50.82)	14 (29.8)	4.447	0.035^*∗*^
Disturbance in respiration	3 (4.92)	3 (6.4)	0.095	0.758
Cough or snoring	13 (19.67)	3 (6.4)	0.22	0.639
Feeling cool	2 (3.29)	2 (4.3)	0.062	0.803
Feeling hot	10 (16.39)	4 (8.5)	1.542	0.241
Nightmares	12 (19.67)	1 (2.1)	7.887	0.005^*∗∗*^
Pain	11 (18.03)	0	9.604	0.002^*∗∗*^

PD: Parkinson's disease; PNS: poor nighttime sleep; PD-PNS: PD with poor nighttime sleep; nPD-PNS: poor nighttime sleep without PD; BMI: body mass index; PSQI: Pittsburgh sleep quality index; ESS: Epworth sleepiness scale; RBD: rapid eye movement (REM) sleep behavior disorder; RLS: restless legs syndrome; HAMA: Hamilton's anxiety scale; HAMD: Hamilton's depression scale. ^*a*^Mann–Whitney test; ^*b*^*t*-test; ^*∗*^*P* < 0.05; ^*∗∗*^*P* < 0.01.

**Table 3:** Correlation coefficients of the PSQI global score and PSQI components with other factors in PD-PNS patients.

	PSQI global score	Subjective sleep	Sleep latency	Sleep duration	Sleep efficiency	Sleep disturbance	Sleep medication	Daytime dysfunction
Age	0.045	0.02	0.119	−0.061	−0.061	−0.009	0.098	0.121
Sex	0.094	0.193	0.280^*∗*^	−0.074	0.110	0.082	0.165	−0.058
BMI	0.216^*∗*^	0.136	0.081	0.177	0.267^*∗*^	0.161	0.171	0.204
Age (*m*)	0.146	0.250^*∗*^	0.078	0.226^*∗*^	0.229^*∗*^	0.203	0.131	0.247^*∗*^
LED (mg)	−0.001	0.148	0.192	0.110	0.158	0.208	0.015	0.132
H&Y (on)	0.223^*∗*^	0.246^*∗*^	0.214	0.110	0.121	0.338^*∗∗*^	−0.032	0.322^*∗∗*^
UPDRS I	0.501^*∗∗*^	0.517^*∗∗*^	0.302^*∗*^	0.552^*∗∗*^	0.480^*∗∗*^	0.511^*∗∗*^	−0.072	0.509^*∗∗*^
UPDRS III	0.425^*∗∗*^	0.306^*∗*^	0.367^*∗∗*^	0.202	0.251	0.363^*∗∗*^	−0.022	0.265
ESS	−0.296^*∗∗*^	−0.363^*∗∗*^	−0.174	−0.335^*∗∗*^	−0.368^*∗∗*^	−0.407^*∗∗*^	−0.171	−0.384^*∗∗*^
RBD	0.227^*∗*^	0.153	0.219	0.188	0.213	0.380^*∗∗*^	0.032	0.159
RLS	0.254^*∗∗*^	0.145	0.064	0.132	0.108	0.134	0.047	0.465^*∗∗*^
HAMD	0.466^*∗∗*^	0.497^*∗∗*^	0.416^*∗∗*^	0.442^*∗∗*^	0.480^*∗∗*^	0.380^*∗∗*^	0.085	0.432^*∗∗*^
HAMA	0.329^*∗∗*^	0.355^*∗∗*^	0.351^*∗∗*^	0.346^*∗∗*^	0.311^*∗∗*^	0.378^*∗∗*^	0.118	0.336^*∗∗*^

BMI: body mass index; LED: daily levodopa-equivalent dose; H&Y: Hoehn–Yahr stage; UPDRS-I: united Parkinson's disease ranking scale part-I; UPDRS III: united Parkinson's disease ranking scale part- III; PSQI: Pittsburgh sleep quality index; ESS: Epworth sleepiness scale; RBD: rapid eye movement (REM) sleep behavior disorder; RLS: restless legs syndrome; HAMA: Hamilton's anxiety scale; HAMD: Hamilton's depression scale; ^*∗*^*P* < 0.05; ^*∗∗*^*P* < 0.01.

**Table 4:** Regression analysis of factors associated with the PSQI global score of PD-PNS patients.

Independent variable	Unstandardized regression coefficient (*B*)	Standard error (SE)	Standardized regression coefficient (*β*)	*t* value	*P* value
Constant term	15.564	2.566	—	6.065	≤0.001
H&Y	4.146	0.908	0.743	4.566	≤0.001
UPDRS-III	0.148	0.035	0.656	4.209	≤0.001
RLS	2.562	1.267	0.208	2.022	0.049
HAMD	0.552	0.131	0.553	4.205	≤0.001

*R* ^2^ = 0.577; final model, adjusted *R*^2^ = 0.541. PSQI: Pittsburgh sleep quality index; PD-PNS: Parkinson's disease with poor nighttime sleep; H&Y: Hoehn–Yahr stage; UPDRS III: united Parkinson's disease ranking scale part III; RLS: restless legs syndrome; HAMD: Hamilton's depression scale.

## Data Availability

The data used to support the findings of this study are included within the article. Requests for data, 12 months after publication of this article, will be considered by the corresponding author.
